# Acrolein-induced atherosclerosis *via* AMPK/SIRT1-CLOCK/BMAL1 pathway and the protection from intermittent fasting

**DOI:** 10.7555/JBR.38.20240025

**Published:** 2024-05-29

**Authors:** Qianfeng Chen, Yuxia Zhong, Bohan Li, Yucong Feng, Yuandie Zhang, Tao Wei, Margaret Zaitoun, Shuang Rong, Hua Wan, Qing Feng

**Affiliations:** 1 Department of Nutrition and Food Hygiene, Key Laboratory of Toxicology, School of Public Health, Nanjing Medical University, Nanjing, Jiangsu 211166, China; 2 Department of Nosocomial Infection Management, Suzhou Wujiang District Children's Hospital, Suzhou, Jiangsu 215299, China; 3 Department of Preventive Medicine, Department of Nutritional Health and Toxicology, School of Public Health, Wuhan University of Science and Technology, Wuhan, Hubei 430065, China; 4 Healthcare Center, Sir Run Run Hospital, Nanjing Medical University, Nanjing, Jiangsu 211112, China

**Keywords:** intermittent fasting, acrolein, circadian rhythm, AMPK, SIRT1, MAPK

## Abstract

The circadian clock is crucial for the progression of cardiovascular diseases. Our previous studies showed that acrolein, an environmental pollutant, exacerbated atherosclerosis by reducing CLOCK/BMAL1 levels and disrupting circadian rhythm; in contrast, intermittent fasting (IF), a dietary regimen, ameliorated acrolein-induced atherosclerosis. In the current study, mice were administered acrolein at a dose of 3 mg/(kg·day) *via* drinking water and subjected to IF for 18 h (from 0:00 to 18:00). We observed that IF reduced the formation of aortic lesions accelerated by acrolein in *ApoE*^−/−^ mice. Upon exposure to acrolein, the expression of *RelA*, *Il1b*, and *Tnf* increased in the liver and heart tissues, but these changes were reversed by IF treatment. Notably, IF treatment upregulated the expression of adenosine monophosphate (AMP)-activated protein kinase catalytic subunit alpha-1 (AMPKα1), p-AMPKα1, and sirtuin 1 (SIRT1), while inhibiting acrolein-induced mitogen-activated protein kinase (MAPK) activation. Additionally, the expression of circadian genes *Clock*/*Bmal1* was suppressed and disrupted by acrolein, whereas IF restored their expression. Moreover, consistent with the *in vivo* findings, short-term starvation *in vitro*, as a fasting cell model, alleviated the dysregulation of CLOCK/BMAL1 and upregulated SIRT1 expression by modulating the AMPK and reactive oxygen species (ROS)-MAPK pathways activated by acrolein. In summary, we demonstrated that IF suppressed the ROS-MAPK pathway but activated the AMPK pathway to enhance the expression of circadian clock genes, thereby ameliorating acrolein-induced atherogenesis, which may shed light on strategies for preventing cardiovascular diseases.

## Introduction

Cardiovascular disease (CVD) remains the leading cause of death worldwide, and atherosclerosis is one of the main pathological bases of CVD. A growing number of studies have indicated that environmental pollution has significant effects on the development of CVD^[1–2]^. Acrolein, an α,β-unsaturated aldehyde commonly found in cigarette smoke, car exhaust, cooking fumes, and food, has been reported to be associated with an increased risk of CVD^[3]^. Studies have shown that acrolein may initiate and accelerate the formation of atherosclerotic lesions through various mechanisms, such as oxidative stress, enhancement of inflammatory processes, and promotion of matrix metalloproteinases^[4]^. Acrolein exposure is thought to induce systemic dyslipidemia, a significant risk factor for the development of atherosclerosis. Furthermore, acrolein increases platelet activation and stimulates the coagulation cascade, leading to thrombosis^[5]^. The prevalence of vascular disease may decrease when acrolein exposure from known sources is reduced. Fortunately, dietary factors like olive extract, asparagus extract, and dietary polyphenols can attenuate acrolein-induced CVD^[6–8]^, suggesting that diet may provide a protective effect against cardiovascular diseases resulting from environmental pollution.

Intermittent fasting (IF) is a dietary regimen that involves alternating periods of fasting and unrestricted eating to prevent and treat diseases such as obesity, type 2 diabetes mellitus, and CVD. Forms of IF include time-restricted feeding (TRF, which limits eating to specific periods), the 5∶2 diet (consisting of five days of normal eating and two days of fasting), and alternate-day fasting (ADF, where one day involves normal eating and the next involves partial fasting)^[9]^. As a dietary pattern, IF does not directly restrict energy intake but rather involves fasting during specific times and unrestricted eating at others, which is considered safer than direct energy restriction^[10]^. Research has shown that IF may lower risk factors associated with atherosclerosis, such as inflammatory markers and blood lipid levels^[11]^. Furthermore, IF has been found to significantly decrease monocyte adhesion and hypercholesterolemia, thereby ameliorating atherosclerosis in *LDLR*^−/−^ mice^[12]^. However, the effects of IF on atherosclerosis and its underlying mechanism remain to be fully elucidated.

Disturbances in circadian rhythms are thought to contribute to the development of CVD^[13]^. Studies have demonstrated that individuals chronically exposed to shift work, night work, jet lag, and sleep disorders are more susceptible to CVD^[14–15]^. The circadian rhythm is regulated by molecular clock genes, including the core clock genes circadian locomotor output cycles protein kaput (*CLOCK*) and aryl hydrocarbon receptor nuclear translocator-like protein 1 (*ARNTL*; also known as *BMAL1*). CLOCK and BMAL1 regulate the expression of the period circadian proteins (*PER1* and *PER2*) and cryptochromes (*CRY1* and *CRY2*) through a transcription-translation negative feedback loop to generate molecular oscillations^[16]^. Deletion or mutation of circadian clock genes results in disturbances to biological rhythms, inflammation, and metabolic diseases^[17]^. It has been reported that *Bmal1* deficiency enhances nuclear factor kappa-B (NF-κB) signaling, oxidative stress, and inflammatory response in mice, suggesting an effect of circadian rhythms on atherosclerosis^[18]^. Our previous studies have shown that acrolein disrupts the circadian clock and activates the mitogen-activated protein kinase (MAPK) related molecules^[6,19]^. However, it remains unclear whether IF can reverse the circadian rhythm disturbance and the upregulation of MAPK caused by acrolein.

IF is closely related to energy-response molecules, such as adenosine monophosphate (AMP)-activated protein kinase (AMPK) and sirtuin 1 (SIRT1). As an energy regulator, AMPK contributes to the pathophysiology of diabetes, cardiometabolic diseases, and cancer^[20]^. Activation of AMPK upregulates nicotinamide phosphoribosyl transferase (NAMPT), thereby increasing intracellular NAD^+^ levels and driving the rhythmic activity of SIRT1. SIRT1 acts as a sensor of nutritional status and circadian rhythm regulation, affecting the circadian clock in the brain and peripheral tissues^[21]^. Recent studies have shown that environmental pollutants induce oxidative stress, which disrupts the circadian clock^[22–23]^. Additionally, MAPK may accelerate atherosclerosis by stimulating the production of reactive oxygen species (ROS)^[24]^. Protein structure analysis has demonstrated that AMPK phosphorylates serine residuals on PERs to modulate CRY stability, while MAPK phosphorylates CRYs at specific regions, thereby attenuating CRY-dependent transcriptional inhibition of the CLOCK/BMAL1 complex^[22]^. Thus, we hypothesized that AMPK-SIRT1 and ROS-MAPK pathways participate in the regulation of the circadian clock through the redox-dependent signaling.

Since IF optimizes feeding times and circadian rhythms in various mammalian organs^[25]^, it remains uncertain whether IF ameliorates atherosclerosis through modulation of AMPK, MAPK, and circadian clock genes. Therefore, we aimed to investigate the effect of IF on acrolein-induced atherosclerosis and its potential molecular mechanisms. We expect this study will provide new insights into the preventive and therapeutic roles of IF in cardiovascular diseases.

## Materials and methods

### Cell culture

Human umbilical vein endothelial cells (HUVECs), murine aortic vascular smooth muscle cells (MOVAS), and RAW264.7 cells were obtained from the Cell Bank of the Chinese Academy of Sciences Committee on Type Culture Collection (Shanghai, China). Cells were cultured in Dulbecco's Modified Eagle Medium (DMEM; Procell, Wuhan, China) containing 10% fetal bovine serum (FBS; HAKATA, Shanghai, China), 100 U/mL penicillin, and 100 μg/mL streptomycin (Beyotime, Shanghai, China) at 37 ℃ in a 5% CO_2_ incubator.

Short-term starvation (STS) was induced by culturing cells in low-glucose, sugar-free DMEM (Procell) supplemented with 0.5 g/L glucose and 1% FBS^[26]^. Because investigators established STS based on a fasting-mimicking diet of 1–2 days per week (energy reduction to 20%–25% on fasting days), we used STS as a cellular model of IF. Specifically, cells were cultured in DMEM high-glucose complete medium in Petri dishes for 2–3 days, and then were switched to the above-mentioned STS solution and cultured for an additional 16–24 h.

### Western blotting

Cells or tissues were lysed in ice-cold RIPA buffer (Beyotime) containing 1% (v/v) phenylmethanesulfonyl fluoride (PMSF; Beyotime). Protein concentrations were determined using the BCA Protein Assay Kit (Beyotime). Equal amounts of protein samples were separated on 8% SDS-polyacrylamide gels and transferred onto polyvinylidene difluoride (PVDF) membranes (Millipore, Boston, MA, USA). After blocking with 5% non-fat milk, the membrane was first incubated with the primary antibody overnight at 4 ℃, followed by incubation with appropriate secondary antibodies. Protein bands were visualized using a horseradish peroxidase (HRP)-based chemiluminescent substrate, and the gray values of target protein bands were quantified using image analysis software (Tanon, Shanghai, China).

Anti-AMPKα1 (1∶1000, Cat. #380431), anti-p-AMPKα1 (1∶1000, Cat. #310044), anti-SIRT1 (1∶1000, Cat. #R25721), anti-p-p38 (1∶1000, Cat. #310068), anti-ERK1/2 (1∶1000, Cat. #R22685), anti-p-ERK1/2 (1∶1000, Cat. #343830), anti-JNK (1∶1000, Cat. #R22866), anti-p-JNK (1∶1000, Cat. #340810), anti-CLOCK (1∶1000, Cat. #R381971), anti-BMAL1 (1∶1000, Cat. #220406), anti-NF-κB (1∶1000, Cat. #222013), anti-IL-6 (1∶1000, Cat. #500286), anti-IL-1β (1∶1000, Cat. #660092), anti-TNF-α (1∶1000, Cat. #346654), anti-p38 (1∶1000, Cat. #R27123), and anti-GAPDH (1∶1000, Cat. #250133) were obtained from ZENBIO (Chengdu, Sichuan, China). Secondary antibodies included HRP-conjugated AffiniPure goat anti-rabbit IgG (1∶2000, Cat. #S0001, Affinity, Alum Creek, WV, USA) and goat anti-mouse IgG (1∶2000, Cat. #S0002, Affinity).

### RNA isolation and real-time reverse transcription-PCR (RT-qPCR)

Total RNA was extracted from the samples using the RNAiso Plus Kit (YIFEIXUE Bio, Nanjing, China) following the manufacturer's instructions. Reverse transcription was performed using Prime Script^TM^ RT Master Mix (Takara, Dalian, China). Real-time PCR was performed using SYBR Premix Ex Taq Ⅱ (YIFEIXUE) and a Roche Light Cycler 96 or 480 Real-Time PCR System (Roche, Basel, Switzerland). Primer sequences are listed in ***Table 1***. Relative gene expression levels were calculated using the 2^−ΔΔCt^ method with *GAPDH* as the internal control.

**Table 1:** Primer sequences used for real-time reverse transcription-PCR

Species	Genes	Forward primers	Reverse primers
Human	*PRKAA1*	5′-GTAGTAAAAACAGGCTCCACGAA-3′	5′-CACCAGAAAGGATCTGTTGGA-3′
Human	*SIRT1*	5′-TGGCAAAGGAGCAGATTAGTAGG-3′	5′-CTGCCACAAGAACTAGAGGATAAGA-3′
Human	*CLOCK*	5′-AAAATACTCTCTACTCATCTGCTGC-3′	5′-ATGGCTCCTTTGGGTCTATTG-3′
Human	*BMAL1*	5′-GCTCCACTGACTACCAAGAA-3′	5′-CTTCCCTTGCATTTTTTATCC-3′
Human	*GAPDH*	5′-CAAGGTCATCCATGACAACTTTG-3′	5′-GTCCACCACCCTGTTGCTGTAG-3′
Mouse	*Prkaa1*	5′-GTCAAAGCCGACCCAATGATA-3′	5′-CGTACACGCAAATAATAGGGGTT-3′
Mouse	*Sirt1*	5′-TGATTGGCACCGATCCTCG-3′	5′-CCACAGCGTCATATCATCCAG-3′
Mouse	*Clock*	5′-ATGGTGTTTACCGTAAGCTGTAG-3′	5′-CTCGCGTTACCAGGAAGCAT-3′
Mouse	*Bmal1*	5′-GACCTACTCTCCGGTTCCCT-3′	5′-ATTTTGTCCCGACGCCTCTT-3′
Mouse	*RelA*	5′-ATGGCAGACGATGATCCCTAC-3′	5′-CGGAATCGAAATCCCCTCTGTT-3′
Mouse	*Il6*	5′-CTGCAAGAGACTTCCATCCAG-3′	5′-AGTGGTATAGACAGGTGTGTTGG-3′
Mouse	*Il1b*	5′-GAAATGCCACCTTTTGACAGTG-3′	5′-TGGATGCTCTCATCAGGACAG-3′
Mouse	*Tnf*	5′-GACGTGGAACTGGCAGAAGAG-3′	5′-TTGGTGGTTTGTGAGTGTGAG-3′
Mouse	*Gapdh*	5′-TGGCCTTCCGTGTTCCTA-3′	5′-GAGTTGCTGTTGAAGTCGCA-3′

### ROS generation assay

DCFH-DA (Beyotime) was diluted 1∶1000 in serum-free medium. The cells were collected and incubated with DCFH-DA at 37 ℃ for 20 min. They were then washed three times with serum-free medium and twice with PBS. Finally, the cells were resuspended in 400 μL of PBS and analyzed using flow cytometry.

### siRNA and plasmid transfection

The siRNAs specific to *PRKAA1* (the gene encoding AMPKα1) or the corresponding control (RiboBio, Guangzhou, China), as well as the *PRKAA1* plasmid or the control plasmid (Beyotime), were transfected into cells using Lipofectamine 2000 (Invitrogen, Carlsbad, CA, USA) according to the manufacturer's instructions. The siRNA sequences targeting *PRKAA1* are listed in ***Table 2***.

**Table 2:** Target sequences of siRNAs for transfection

Species	siRNAs	Sequences (5′-3′)
Human	si*PRKAA1*-01	GUGGAACCCUUCCAUUUGA
Human	si*PRKAA1*-02	GAUCCAUCAUAUAGUUCAA
Human	si*PRKAA1*-03	ACAGGAGAAUAAUGAAUGA
Mouse	si*Prkaa1*-01	UUGCUCUACAAAGUUUGCU
Mouse	si*Prkaa1*-02	UAGAUUUGCACACAUUUCC
Mouse	si*Prkaa1*-03	UUGAACUAUAAGAUGGGUC

### *In vivo* studies

Twenty male *ApoE*^−/−^ mice (6–7 weeks old, 20–25 g) were purchased from the Model Animal Research Center of Nanjing University (Nanjing, Jiangsu, China). Mice were fed a high-fat diet (18.9% protein, 44.6% carbohydrate, and 36.5% fat) for eight weeks at the Laboratory Animal Center of Nanjing Medical University to establish atherosclerosis models, and then divided into four groups that were fed under a 12 h light/12 h dark schedule for eight weeks as follows: the control group was fed freely without acrolein (Sinopharm Chemical Reagent Company, Nanjing, Jiangsu, China); the TRF group was fed for 6 h a day (18:00 to 24:00); the acrolein (ACR) group was fed 3 mg/kg acrolein in drinking water daily; and the acrolein combined with time-restricted feeding (ACR + TRF) group was fed for 6 h (18:00 to 24:00) and given 3 mg/kg acrolein in drinking water daily. Afterward, the mice were sacrificed to collect the aorta, liver, and heart tissues.

Sixty-four C57BL/6J mice, purchased from the Nanjing Medical University Animal Core (Nanjing, Jiangsu, China), were fed a high-fat diet for eight weeks, after which they were randomly divided into four groups and treated for 12 weeks as follows: the control group had free access to food without acrolein; the TRF group was fed for 6 h daily (from 18:00 to 24:00); the ACR group received 3 mg/kg acrolein in drinking water daily; and the ACR + TRF group was fed for 6 h daily (from 18:00 to 24:00) and received 3 mg/kg acrolein in drinking water daily. Mice were then sacrificed at 06:00 (zeitgeber time [ZT] 0/24), 12:00 (ZT6), 18:00 (ZT12), and 24:00 (ZT18), and the aorta, liver, and heart tissues were collected.

Animal experiments were performed in accordance with the Guide for the Care and Use of Laboratory Animals and were approved by the Animal Ethics and Welfare Committee of Nanjing Medical University (Approval No. IACUC-2205022).

### Oil Red O staining

Aortic tissues were dissected and fixed in 4% paraformaldehyde for 48 h. Subsequently, the aortic sections were immersed in the prepared Oil Red O working solution. After staining, the sections were differentiated in 60% isopropanol until the plaques appeared orange and the rest of the normal aorta tissue turned milky white, after which they were photographed. RAW264.7 cells were fixed in 4% paraformaldehyde for 20–30 min, rinsed, and placed into the prepared Oil Red O working solution. Finally, the cells were washed to remove excess dye and photographed using a CKX41 inverted microscope (Olympus, Japan). Image-Pro Plus software (version 6.0; Cybernetics of Media, Bethesda, MD, USA) was used to quantify the staining.

### Serum lipid detection

The concentrations of plasma triglycerides, high-density lipoprotein cholesterol (HDL-C), low-density lipoprotein cholesterol (LDL-C), and total cholesterol were determined using commercial colorimetric kits (Biolabo SAS, Maizy, France) based on glycerol-3-phosphate oxidase (GPO) and cholesterol oxidase phenol 4-aminoantipyrine peroxidase (CHOD-PAP) methods, coupled with enzymatic reactions. The reaction end products were detected by spectrophotometry using a CLARIOstar Plus microplate reader (BMG Labtech, Ortenberg, Germany).

### Statistical analysis

Data are presented as the mean ± standard deviation of three independent experiments. A two-tailed Student's *t-*test was used for comparisons between two groups, while one-way analysis of variance (ANOVA) followed by the Bonferroni multiple comparison test was applied for comparisons among three or more groups. The two-way ANOVA followed by Dunnett's test was applied to compare data between two or more groups under different conditions. All statistical analyses were performed using GraphPad Prism 7 (GraphPad Software, La Jolla, CA, USA). *P* < 0.05 was considered statistically significant.

## Results

### IF attenuated acrolein-accelerated aortic plaque area and inflammatory factors in *ApoE*^−/− ^mice

**Figure 1:**
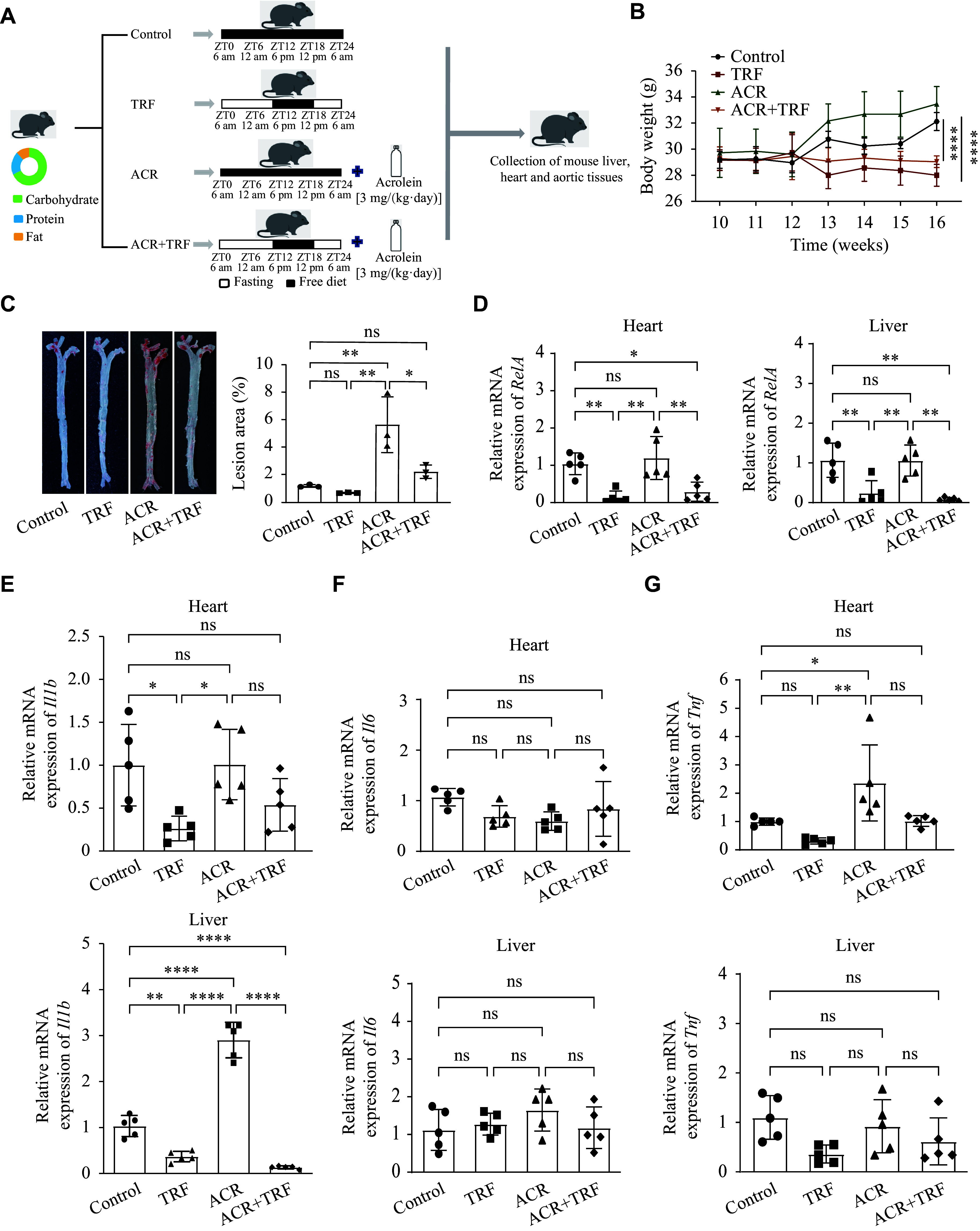
Effects of ACR and/or TRF on atherosclerosis in *ApoE*^−/−^ mice. A: A total of 20 *ApoE*^−/−^ mice, aged 6–7 weeks, were fed a high-fat diet for eight weeks to establish a model of atherosclerosis. They were then divided into four groups (*n* = 5) as follows: the control group (Control), the time-restricted feeding group (TRF) in which mice were fed for 6 h per day (between 18:00 and 24:00), the acrolein group (ACR) in which mice were fed 3 mg/(kg·day) of acrolein by drinking water, and the combined acrolein with time-restricted feeding group (ACR + TRF). All mice were fed a high-fat diet for another eight weeks. B: Body weight of mice in the four groups from weeks 10 to 16 (*n* = 5). C: The aorta tissues of *ApoE*^−/−^ mice were stained with Oil Red O (*n* = 3). Representative images of the aorta of *ApoE*^−/−^ mice were observed by microscope, and the percentage of stained area after treatment with ACR and/or TRF for eight weeks was quantified. D–G: Real-time reverse transcription-PCR was performed to detect the mRNA levels of *RelA* (D), *Il1b* (E), *Il6* (F), and *Tnf* (G) in the heart and liver tissues of the *ApoE*^−/−^ mice (*n* = 5). *Gapdh* served as the internal reference. Genes *RelA*, *Il1b*, *Il6*, and *Tnf* encode nuclear factor kappa B p65 (NF-κB p65), interleukin 1 beta (IL-1β), interleukin 6 (IL-6), and tumor necrosis factor-alpha (TNF-α), respectively. Data are expressed as mean ± standard deviation of at least three experiments and analyzed by one-way analysis of variance (ANOVA), followed by Bonferroni multiple comparison test, except that statistical analysis for panel B was performed by two-way ANOVA followed by Dunnett's tests. ^*^P < 0.05, ^**^P < 0.01, and ^****^P < 0.0001. Abbreviation: ns, not significant.

To investigate whether IF attenuates acrolein-induced development of atherosclerosis, we divided the *ApoE*^−/−^ mice into four groups that were fed a high-fat diet (***Fig. 1A***). The body weight of mice in both the TRF and ACR + TRF groups was significantly lower than that in the control group (***Fig. 1B***). As shown in ***Supplementary Fig. 1A***–***1D*** (available online), ACR markedly increased serum total cholesterol and LDL-C levels, whereas the combined ACR and TRF treatment reversed these increases. Moreover, TRF alone elevated HDL-C levels, but the ACR + TRF combination reduced HDL-C compared with TRF alone. Similarly, the combined ACR + TRF treatment reversed the ACR-induced elevation in TG levels. In the ACR group, the Oil Red-stained aortic plaque area was significantly larger than that in the control group (***Fig. 1C***). Given that inflammation contributes to the progression of atherosclerosis^[27]^, we next examined the mRNA levels of inflammatory markers. *RelA* and *Il1b* expression levels in the liver and heart tissues were significantly decreased in the TRF group, compared with the control group. Additionally, both *RelA* and *Il1b* levels were further reduced in the ACR + TRF group, compared with ACR alone (***Fig. 1D*** and ***1E***). After ACR exposure, only *Il1b* expression levels in the liver and *Tnf* expression levels in the heart were significantly upregulated, compared with the control group (***Fig. 1E*** and ***1G***). There was also a trend toward increased mRNA levels of *Il6*, although this did not reach statistical significance (***Fig. 1F***). Taken together, these findings suggest that IF inhibits acrolein-induced aortic plaque formation and inflammation.

### TRF enhanced the expression of AMPK and SIRT1 but inhibited MAPK induced by ACR in *ApoE*^−/−^ mice

**Figure 2:**
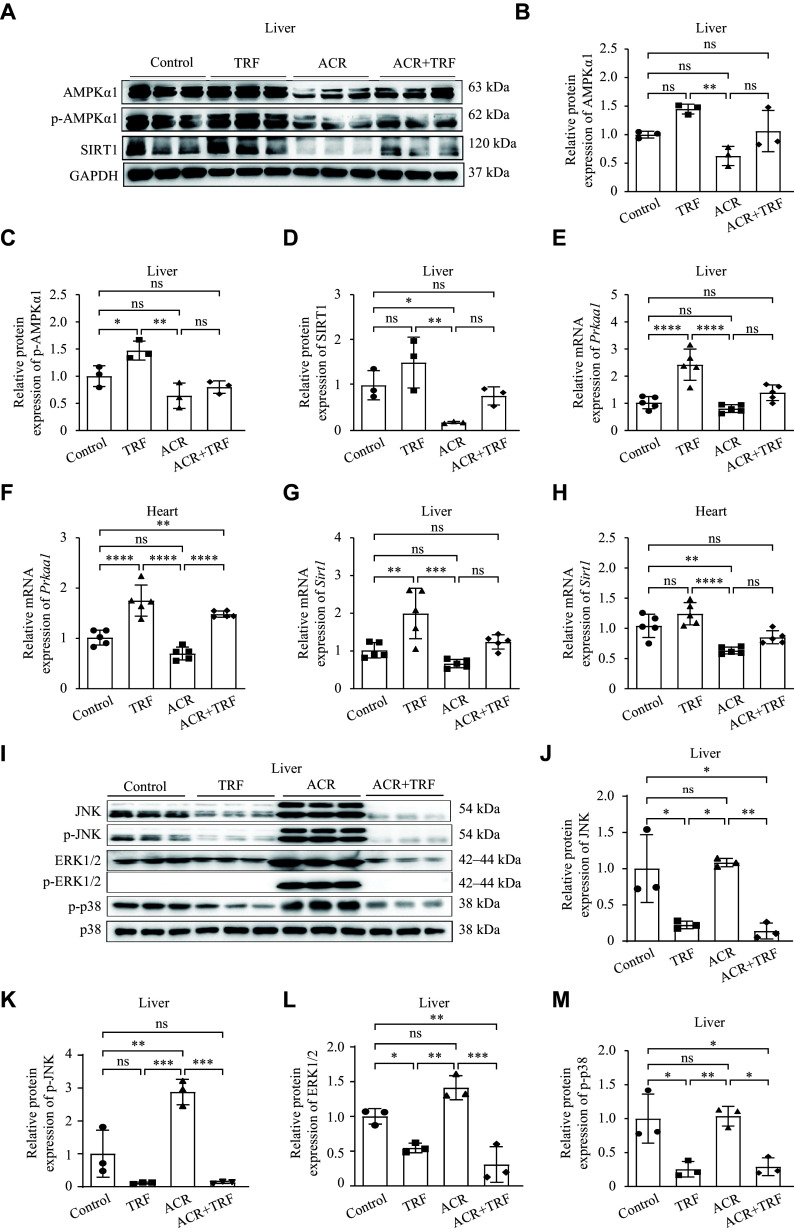
Expression of AMPKα1, SIRT1, and MAPKs after the indicated treatment in *ApoE*^−/−^ mice. The *ApoE*^−/−^ mice were treated with acrolein (ACR) and/or time-restricted feeding (TRF) for eight weeks. A: The protein levels of AMPKα1, p-AMPKα1, and SIRT1 in the liver of *ApoE*^−/−^ mice (*n* = 3) were detected by Western blotting (WB). B–D: The semi-quantitative analyses of AMPKα1 (B), p-AMPKα1 (C), and SIRT1 (D) protein levels were performed, using GAPDH as the reference (*n* = 3). E–H: Real-time reverse transcription-PCR (RT-qPCR) was performed to detect the mRNA levels of *Prkaa1* (the gene encoding AMPKα1; E and F) and *Sirt1* (G and H) in the liver and heart tissues of *ApoE*^−/−^ mice (*n* = 5). I: WB was performed to detect protein levels of JNK, p-JNK, ERK, p-ERK, and p-p38 in the liver of *ApoE*^−/−^ mice (*n* = 3). J–M: The semi-quantitative analyses of JNK (J), p-JNK (K), ERK1/2 (L), and p-p38 (M) protein levels were performed, using p38 as a reference (*n* = 3). Data are expressed as mean ± standard deviation of at least three experiments and analyzed by one-way ANOVA, followed by the Bonferroni multiple comparison test. ^*^P < 0.05, ^**^P < 0.01, ^***^P < 0.001, and ^****^P < 0.0001. Abbreviations: AMPKα1, AMP-activated protein kinase catalytic subunit alpha-1; p-AMPKα1, phosphorylated AMPKα1; SIRT1, sirtuin 1; GAPDH, glyceraldehyde-3-phosphate dehydrogenase; MAPKs, mitogen-activated protein kinases; JNK, c-Jun N-terminal kinase; p-JNK, phosphorylated JNK; ERK1/2, extracellular regulated protein kinase 1/2; p-ERK1/2, phosphorylated ERK; p38, p38 mitogen-activated protein kinase; p-p38, phosphorylated p38; ns, not significant.

It has been shown that the liver is the largest energy-metabolizing organ in the human body^[28]^, and energy-responsive molecules are typically activated during nutrient deprivation or fasting^[29]^. Therefore, we assessed energy-related proteins in *ApoE*^−/−^ mice after the indicated treatments. We found that the protein levels of AMPK, p-AMPK, and SIRT1 in the liver were reduced after ACR treatment but increased after TRF treatment, compared with the control group (***Fig. 2A***–***2D***), although the differences were not significant. Moreover, compared with the ACR group, the ACR + TRF group displayed an upward trend in these energy indicators. Meanwhile, the same trend was observed at the mRNA levels in both liver and heart tissues (***Fig. 2E***–***2H***). These results suggest that IF may ameliorate the suppressive effects of acrolein on energy-sensing molecules such as AMPK and SIRT1.

Our previous studies have demonstrated that acrolein activates the MAPK pathway^[6,19]^. To further explore, we assessed hepatic MAPK pathway proteins in *ApoE*^−/−^ mice after the indicated conditions. Compared with the control group, TRF significantly decreased the protein levels of JNK, p-JNK, and p-p38. Notably, acrolein treatment did not significantly increase these protein levels compared with the control group, except for ERK1/2 (***Fig. 2I***–***2M***). Moreover, the expression levels of MAPK pathway proteins were significantly down-regulated in the ACR + TRF group, compared with the ACR group (***Fig. 2J***–***2M***). These results indicate that IF suppresses acrolein-induced MAPK activation in *ApoE*^−/−^ mice.

### TRF alleviated acrolein-induced circadian rhythm disturbances and restored *Clock*/*Bmal1* expression in mice

**Figure 3:**
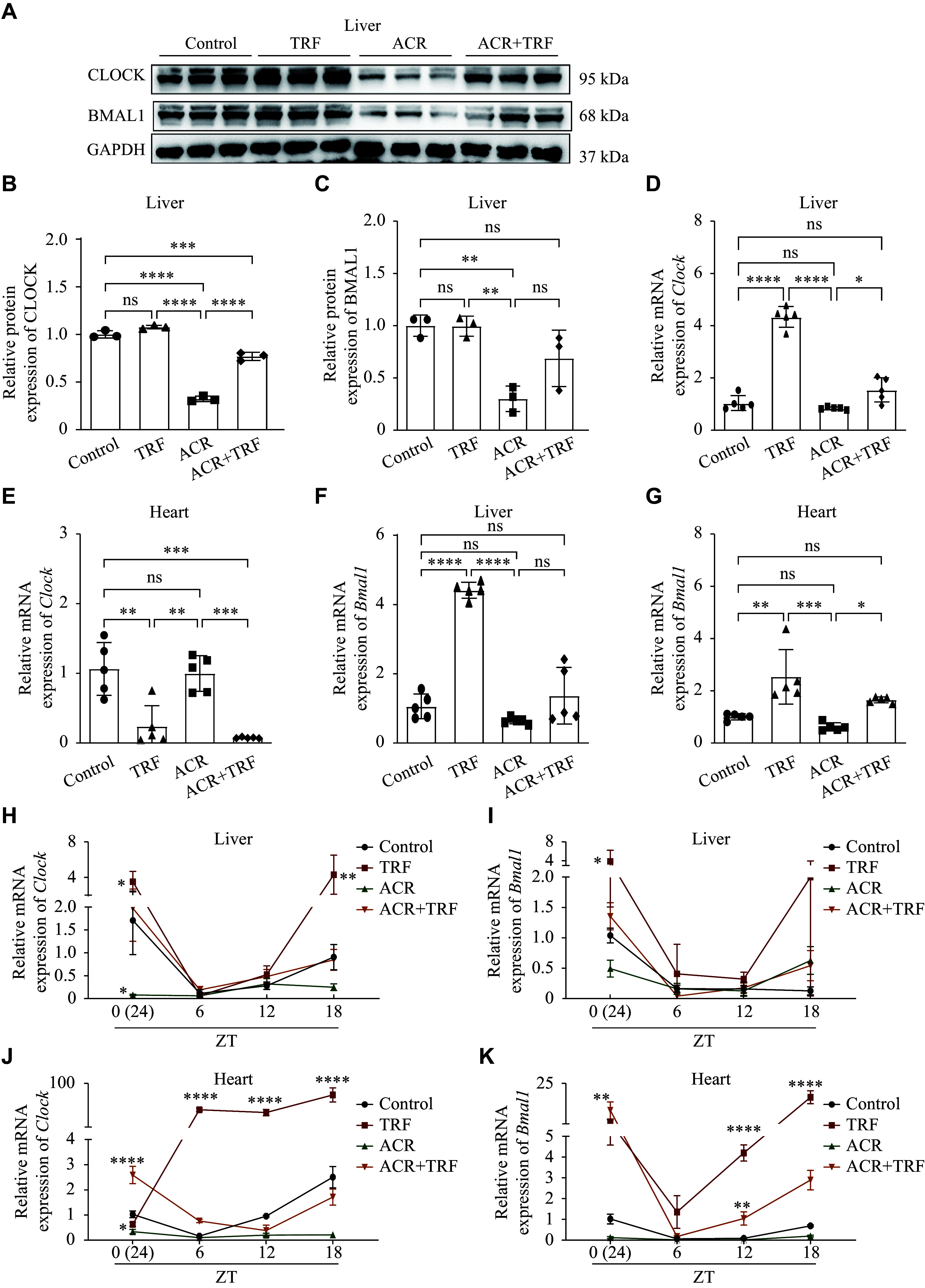
Expression of CLOCK and BMAL1 in the mice treated with ACR and/or TRF. Twenty *ApoE*^−/−^ mice were treated with acrolein (ACR) and/or time-restricted feeding (TRF) for eight weeks. A: The protein levels of CLOCK and BMAL1 in the liver of *ApoE*^−/−^ mice (*n* = 3) were detected by Western blotting. B and C: The semi-quantitative analyses of CLOCK (B) and BMAL1 (C) protein levels were performed, using GAPDH as a reference (*n* = 3). D–G: The mRNA levels of *Clock* (D and E) and *Bmal1* (F and G) in the liver and heart tissues of *ApoE*^−/−^ mice (*n* = 5) were detected by quantitative reverse transcription-PCR (RT-qPCR). H–K: The mRNA levels of *Clock* (H and J) and *Bmal1* (I and K) in the liver and heart tissues at 6:00 (ZT0), 12:00 (ZT6), 18:00 (ZT12), and 24:00 (ZT18) of 64 C57BL/6J mice (*n* = 4) were detected by RT-qPCR. Data are expressed as mean ± standard deviation of at least three experiments and analyzed by ANOVA, followed by the Bonferroni multiple comparison test. ^*^P < 0.05, ^**^P < 0.01, ^***^P < 0.001, and ^****^P < 0.0001 compared with the control group. Abbreviations: GAPDH, glyceraldehyde-3-phosphate dehydrogenase; Bmal1, brain and muscle ARNT-like 1; Clock, circadian locomotor output cycles kaput; ns, not significant; ZT, zeitgeber time.

As the liver is also the largest peripheral circadian organ^[30]^, we examined the expression of circadian clock genes in the heart and liver tissues of *ApoE*^−/−^ mice. As shown in ***Fig. 3A***–***3C***, ACR exposure significantly decreased CLOCK and BMAL1 levels in the liver. Additionally, TRF up-regulated *Bmal1* expression in both heart and liver tissues (***Fig. 3F*** and ***3G***), whereas *Clock* expression was elevated only in the liver (***Fig. 3D***). Compared with the ACR group, *Clock* and *Bmal1* expression levels were partially restored by TRF in the ACR + TRF group, showing an upward trend in both the liver and heart (***Fig. 3D*** and ***3G***). Taken together, these results indicate that the ameliorative effects of TRF on ACR-induced changes may be related to the improvements in circadian clock function.

"Zeitgeber" refers to any environmental cue that synchronizes 24-h rhythmic behavior. To examine the effect of IF on circadian rhythm, we assessed the temporal expression patterns of core clock genes in *ApoE*^−/−^ mice under the indicated treatments. The results showed that the expression levels of *Clock* and *Bmal1* were lower in the heart and liver during the daytime (ZT6 or ZT12), then increased to higher levels at night (ZT18 or ZT24/0) (***Fig. 3H***–***3K***). The rhythmicity of the TRF group was consistent with that of the control group, with a significant nighttime increase in *Clock* and *Bmal1* expression. However, the rhythmical expression pattern of *Clock* and *Bmal1 *was disrupted in the ACR group. Notably, TRF restored the acrolein-induced suppression of *Clock* and *Bmal1* expression (***Fig. 3H***–***3K***). These results indicate that IF alleviates acrolein-induced circadian rhythm disturbances.

### Short-term starvation (STS) suppressed acrolein-stimulated cell foam and ROS-MAPK

**Figure 4:**
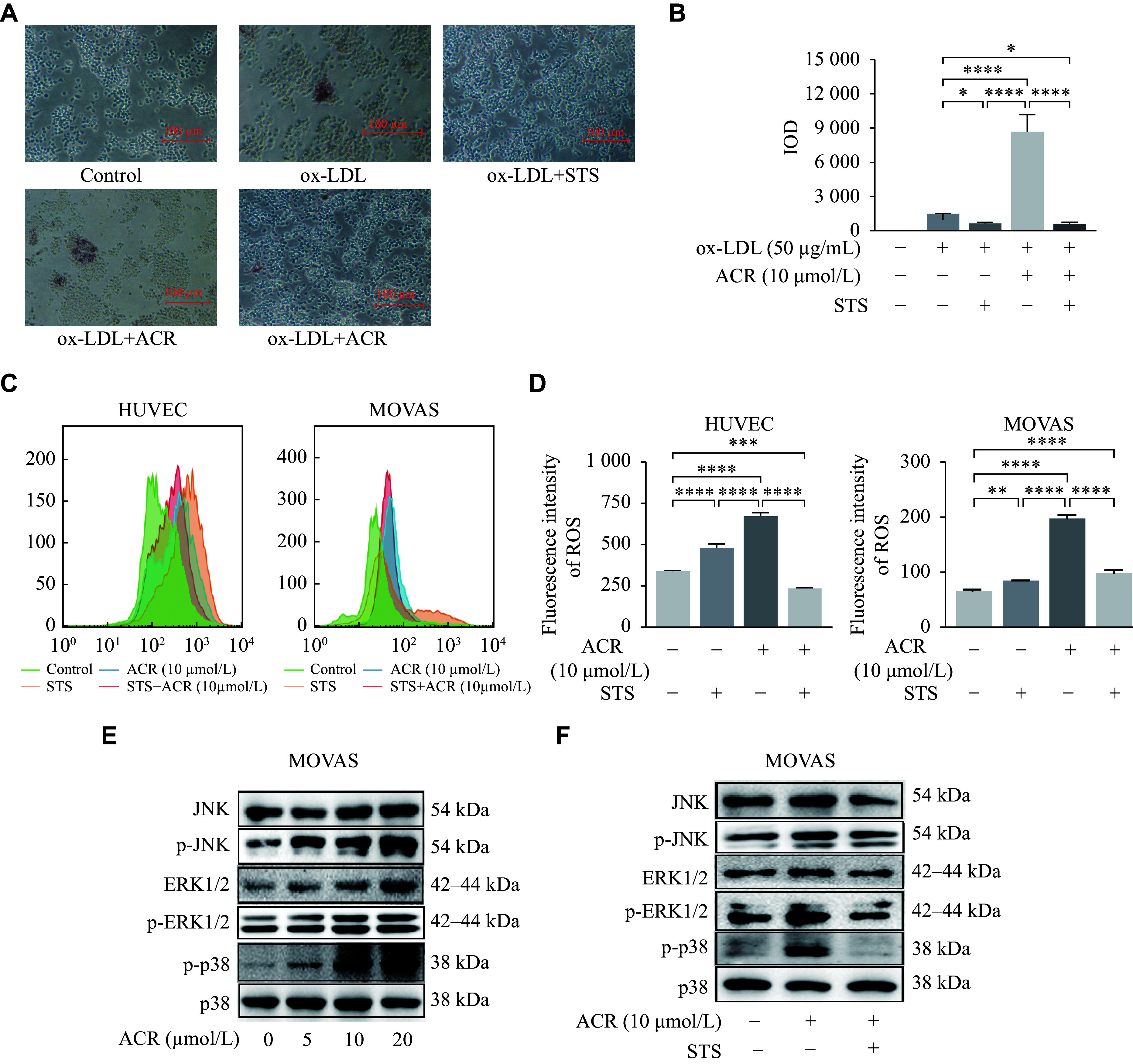
Effects of ACR and/or STS on cell lipid droplet uptake, ROS, and MAPK expression in cells. A: RAW264.7 cells were treated with acrolein (ACR; 10 μmol/L) and/or short-term starvation (STS) and oxidized low-density lipoprotein (ox-LDL; 50 μg/mL) for 24 h. The cells were stained with Oil Red O and photographed under a microscope. Scale bars, 100 μm. B: The semi-quantitative analyses of lipid droplet uptake in panel A were performed. C: ROS detection by flow cytometry in HUVECs and MOVAS treated with ACR and/or STS for 24 h. D: The semi-quantitative analyses of ROS in panel C were performed. E: Following a 24-h exposure to ACR (0, 5, 10, 20 μmol/L), the protein levels of JNK, p-JNK, ERK1/2, p-ERK1/2, and p-p38 in MOVAS were determined by Western blotting (WB), using p38 as a reference. F: The protein levels of JNK, p-JNK, ERK1/2, p-ERK1/2, and p-p38 in MOVAS treated with ACR (10 μmol/L) and/or STS for 24 h were determined by WB, using p38 as a reference. Data for the semi-quantitative analyses of protein levels (E and F) are shown in ***Supplementary Fig. 2***. Data are expressed as mean ± standard deviation of at least three experiments and analyzed by one-way ANOVA, followed by the Bonferroni multiple comparison test. ^*^P < 0.05, ^**^P < 0.01, ^***^P < 0.001, and ^****^P < 0.0001 (*n* = 3). Abbreviations: ROS, reactive oxygen species; MAPKs, mitogen-activated protein kinases; JNK, c-Jun N-terminal kinase; p-JNK, phosphorylated c-Jun N-terminal kinase; ERK1/2, extracellular regulated protein kinase 1/2; p-ERK1/2, phosphorylated ERK1/2; p38, p38 mitogen-activated protein kinase; p-p38, phosphorylated p38; HUVECs, human umbilical vein endothelial cells; MOVAS, murine aortic vascular smooth muscle cells.

To further explore the mechanism by which TRF ameliorates the atherosclerosis triggered by acrolein, we used STS as a cell model of IF. ACR exposure increased the uptake of lipid droplets in macrophages, whereas STS reversed this effect (***Fig. 4A*** and ***4B***), indicating that STS can reduce macrophage foam cell formation.

Because the ROS-MAPK pathway plays a critical role in atherogenesis, we measured ROS levels in endothelial and smooth muscle cells. As shown in ***Fig. 4C*** and ***4D***, STS alone induced a slight increase in ROS, while ACR significantly increased ROS levels. However, this elevation was inhibited when STS was present. Moreover, the protein levels of JNK, p-JNK, ERK1/2, p-ERK1/2, and p-p38 were elevated in cells treated with various doses of ACR (***Fig. 4E***, ***Supplementary Fig. 2A***–***2E*** [available online]). Conversely, the protein levels of MAPK-related molecules were reduced by STS treatment (***Fig. 4F***, ***Supplementary Fig. 2F***–***2J ***[available online]). These findings indicate that STS can inhibit both ROS and MAPK activation stimulated by ACR.

### STS reversed the acrolein-induced downregulation of AMPK, SIRT1, and CLOCK-BMAL1 in HUVECs and MOVAS

**Figure 5:**
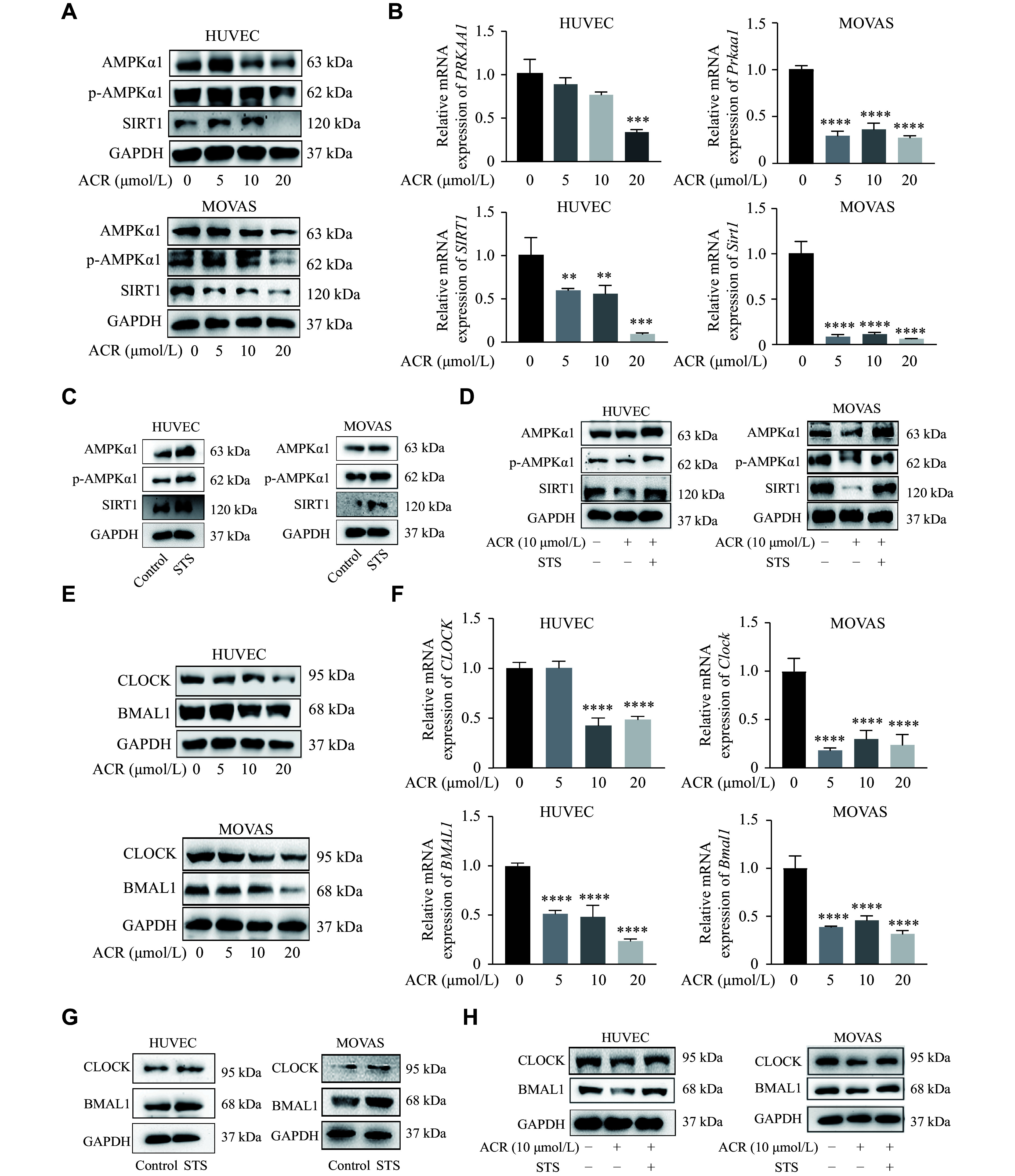
Effects of acrolein and/or STS on expression of AMPKα1, SIRT1, CLOCK, and BMAL1 in HUVECs and MOVAS. A and B: Following various doses of acrolein (ACR; 5, 10, 20 μmol/L) treatment for 24 h, the protein levels of AMPKα1, p-AMPKα1, and SIRT1 in HUVECs and MOVAS were determined by Western blotting (WB; A), while the mRNA levels of *AMPK* and *SIRT1* were determined by real-time reverse transcription-PCR (RT-qPCR; B). C: The protein levels of AMPKα1, p-AMPKα1, and SIRT1 in HUVECs and MOVAS that underwent short-term starvation (STS) for 24 h were determined by WB. D: The protein levels of AMPKα1, p-AMPKα1, and SIRT1 in HUVECs and MOVAS treated with ACR (10 μmol/L) and/or STS for 24 h were determined by WB. E and F: The protein (E) and mRNA (F) levels of *CLOCK* and *BMAL1* in HUVECs and MOVAS were determined by WB and RT-qPCR, respectively, after various doses of ACR (5, 10, 20 μmol/L) treatment for 24 h. G: The protein levels of CLOCK and BMAL1 in HUVECs and MOVAS that underwent STS for 24 h were determined by WB. H: The protein levels of CLOCK and BMAL1 in HUVECs and MOVAS treated with ACR (10 μmol/L) and/or STS for 24 h were determined by WB. Data for the semi-quantitative analyses of protein levels (A, C, D) and (E, G, H) are shown in ***Supplementary Fig. 3*** and ***Supplementary Fig. 4***, respectively. Data are expressed as mean ± standard deviation of at least three experiments. Statistical analysis was performed using one-way ANOVA followed by the Bonferroni multiple comparison test. ^**^P < 0.01, ^***^P < 0.001, and ^****^P < 0.0001. Abbreviations: AMPKα1, AMP-activated protein kinase catalytic subunit alpha-1; p-AMPKα1, phosphorylated AMPKα1; SIRT1, sirtuin 1; GAPDH, glyceraldehyde-3-phosphate dehydrogenase; BMAL1, brain and muscle ARNT-like 1; CLOCK, circadian locomotor output cycles kaput; HUVECs, human umbilical vein endothelial cells; MOVAS, murine aortic vascular smooth muscle cells.

The mRNA and protein levels of AMPK, p-AMPK, and SIRT1 decreased when HUVECs and MOVAS were treated with the indicated doses of acrolein (***Fig. 5A*** and ***5B***, ***Supplementary Fig. 3A*** and ***3B*** [available online]), whereas they increased significantly when the cells were treated with STS (***Fig. 5C***, ***Supplementary Fig. 3C*** and ***3D ***[available online]). Additionally, compared with the ACR group, the expression levels of AMPK, p-AMPK, and SIRT1 were increased after ACR plus STS treatment (***Fig. 5D***, ***Supplementary Fig. 3E*** and ***3F***). Furthermore, the expression levels of protein and mRNA of CLOCK and BMAL1 were reduced by various doses of ACR (***Fig. 5E*** and ***5F***, ***Supplementary Fig. 4A*** and ***4B*** [available online]). However, when STS was applied, both CLOCK and BMAL1 were upregulated (***Fig. 5G***, ***Supplementary Fig. 4C*** and ***4D ***[available online]). Compared with the ACR group, CLOCK and BMAL1 were upregulated in the ACR + STS group (***Fig. 5H***, ***Supplementary Fig. 4E*** and ***4F ***[available online]). These results indicate that IF may reverse the acrolein-induced downregulation of AMPK, SIRT1, and circadian clock genes, thereby alleviating acrolein-accelerated atherosclerosis. The results are consistent with those in the animal study.

### AMPK regulated *SIRT1* and *CLOCK*/*BMAL1* in HUVECs and MOVAS

**Figure 6:**
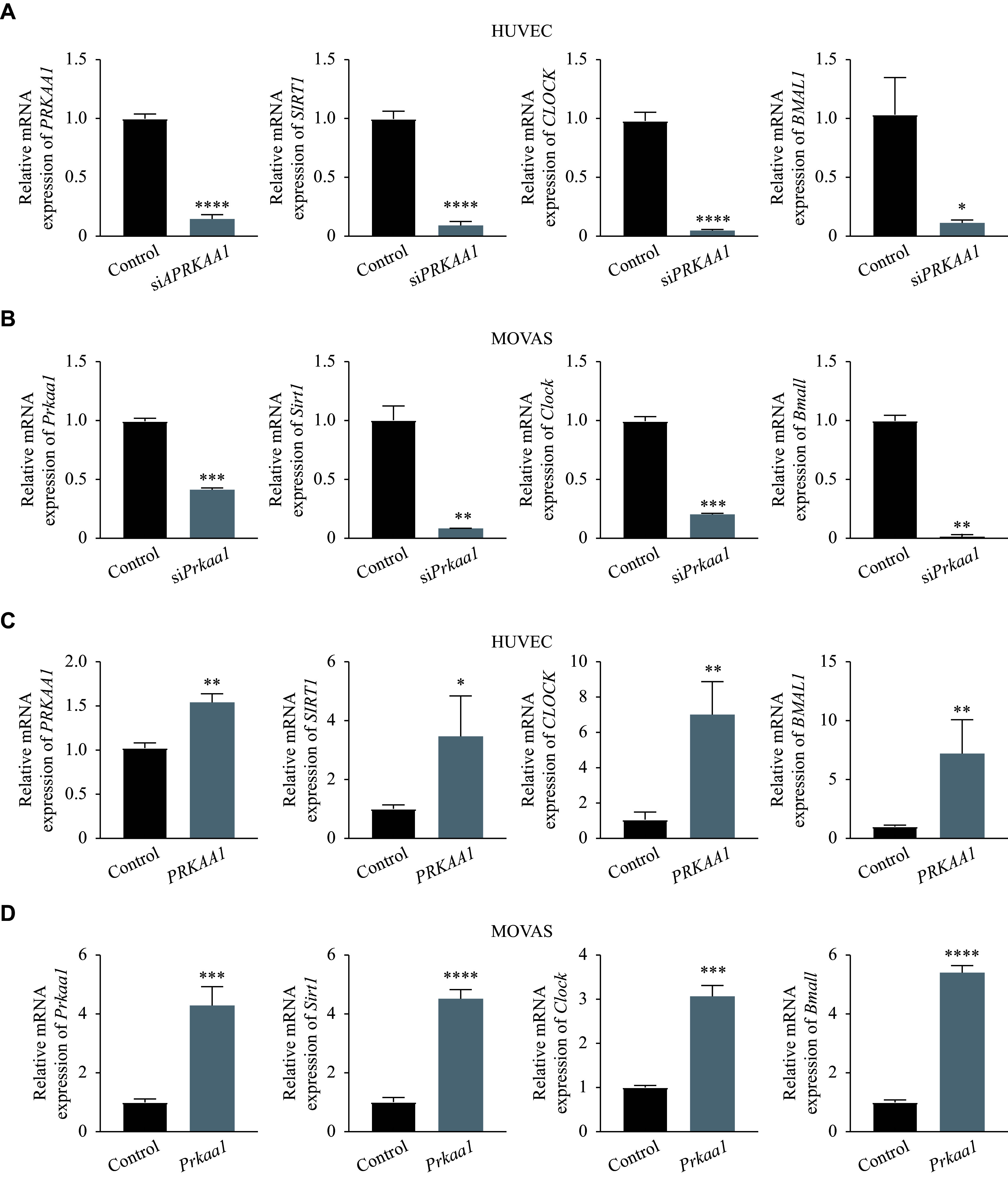
AMPK regulated *CLOCK* and *BMAL1* expression in HUVECs and MOVAS. A: Following the si*PRKAA1* treatment for 24 h, the mRNA levels of *PRKAA1* (the gene encoding AMPKα1), *SIRT1*, *CLOCK*, and *BMAL1* in HUVECs were detected by real-time reverse transcription-PCR (RT-qPCR). B: Following the si*Prkaa1* treatment for 24 h, the mRNA levels of *Prkaa1*, *Sirt1*, *Clock*, and *Bmal1* in MOVAS were detected by RT-qPCR. C: HUVECs were transfected with *PRKAA1* plasmid and cultured for 24 h. The mRNA levels of *PRKAA1*, *SIRT1*, *CLOCK*, and *BMAL1* were detected by RT-qPCR. D: MOVAS were transfected with *Prkaa1* plasmid and cultured for 24 h. The mRNA levels of *Prkaa1*, *Sirt1*, *Clock*, and *Bmal1* were detected by RT-qPCR. Data were expressed as mean ± standard deviation of at least three experiments and analyzed by the two-tailed Student's *t-*test. ^*^P < 0.05, ^**^P < 0.01, ^***^P < 0.001, and ^****^P < 0.0001. Abbreviations: AMPKα1, AMP-activated protein kinase catalytic subunit alpha-1; p-AMPKα1, phosphorylated AMPKα1; SIRT1, sirtuin 1; GAPDH, glyceraldehyde-3-phosphate dehydrogenase; BMAL1, brain and muscle ARNT-like 1; CLOCK, circadian locomotor output cycles kaput; HUVECs, human umbilical vein endothelial cells; MOVAS, murine aortic vascular smooth muscle cells.

We further investigated whether AMPK regulated the expression of SIRT1 and CLOCK/BMAL1 in HUVECs and MOVAS. As shown in ***Fig. 6A*** and ***6B***, we observed that the mRNA levels of *SIRT1* and *CLOCK*/*BMAL1* significantly decreased upon *PRKAA1* knockdown. Conversely, the mRNA levels of *SIRT1, CLOCK*/*BMAL1* were significantly upregulated by *PRKAA1* overexpression (***Fig. 6C*** and ***6D***). Therefore, our data demonstrate that AMPK directly regulates the expression of *SIRT1* and *CLOCK/BMAL1*.

## Discussion

Recently, IF has been demonstrated to function as a dietary regimen that regulates the circadian clock, aligning feeding schedules with the optimal circadian rhythms of various mammalian organs, thereby decreasing the risk of cardiovascular disease^[31]^. Our previous studies demonstrated that acrolein reduced CLOCK/BMAL1 levels and induced circadian rhythm disturbance, while dietary factors were able to ameliorate these effects^[6,19]^. In the current study, we found that IF suppressed acrolein-induced inflammation and atherosclerotic plaque formation. Moreover, IF alleviated the reduction of AMPK, SIRT1, CLOCK, and BMAL1 levels and restored the circadian rhythm disruption caused by ACR exposure in mice. Consistent with the *in vivo* experiment, STS treatment decreased MAPK activation in MOVAS. In addition, STS inhibited acrolein-stimulated ROS and cellular foam formation. To our knowledge, this is the first report of IF as an intervention strategy for pollution-induced atherosclerosis, acting through the AMPK-SIRT1/ROS-MAPK/CLOCK-BMAL1 pathway, thereby offering new insights into the mechanisms of atherosclerosis.

Acrolein induced and accelerated atherosclerosis by promoting LDL-C-induced macrophage foam formation, stimulating ROS production^[32–34]^, triggering the release of inflammatory factors such as IL-1β, IL-6, and TNF-α in HUVECs^[35]^, as well as directly exacerbating the formation of atherosclerotic lesions in the aortic valve and aortic arch^[32,36]^. Consistent with the previous reports^[6,19]^, the current study demonstrated that ACR downregulated circadian clock genes and accelerated the development of atherosclerosis. Additionally, we found that ACR inhibited energy-responsive molecules, AMPK-SIRT1, thereby promoting atherogenesis.

AMPK is involved in anti-atherogenic effects, such as modulating inflammatory factors and reducing foam cell formation. A recent study has reported that SIRT1 protects against inflammation and atherosclerotic plaque development^[37]^. Furthermore, AMPK and SIRT1 are closely linked to circadian rhythms, and fasting activates AMPK^[38–39]^. Our results showed that the expression levels of *SIRT1*, *CLOCK*, and *BMAL1* exhibited parallel changes following *AMPK* knockdown or overexpression, suggesting that IF may exert its anti-atherosclerotic effects by regulating CLOCK and BMAL1 through AMPK.

MAPK plays an important role in the progression of atherosclerosis, as demonstrated in the current study following stimulation by ACR. In addition, p38 MAPK is known to participate in inflammation in different environments and cell types^[40]^. Several recent studies have demonstrated that ROS and inflammatory factors can activate MAPK, thereby influencing the regulation of circadian clock genes^[21,41–42]^. Our findings indicate that IF can reduce ROS and MAPK activation and reverse the circadian rhythm disorder induced by ACR.

The asynchrony between circadian rhythms and metabolism significantly increases the risk of cardiovascular diseases^[16]^. In addition, IF optimizes circadian rhythms and reduces the risk of metabolic disorder^[25]^. We further found that the rhythmicity of IF upregulated the expression of *Clock* and *Bmal1* genes. Interestingly, our data showed that the *Clock* mRNA levels were lower in the heart than in the liver and returned to baseline levels during the day. This is likely because the liver is the largest peripheral organ of biological clock^[21]^, and the oscillation of the circadian rhythm may influence *Clock* gene expression.

Studies have reported that caloric restriction involves a reduction of energy intake without malnutrition^[43]^, while TRF, an alternative feeding method of caloric restriction, restricts daily eating to a period ranging from 4–12 h^[44]^. In our experiments, mice in the TRF group were fed freely between 18:00 and 24:00. Consequently, CLOCK-BMAL1 was upregulated by TRF, not caloric restriction, which was in line with the findings of Jamshed *et al*^[45]^. *In vitro*, a 24-h STS did not cause severe physiological effects, but protected normal fibroblasts by regulating the cell cycle^[46]^. Consistent with our findings, during the early stage of atherosclerosis, IF can reduce aortic plaque formation and increase HDL-C levels^[47]^. However, *Il6* expression was elevated in mice treated with TRF, and a recent study has demonstrated that IF may increase inflammatory factors in *Ldl*^−/−^ mice^[48]^. In the intermediate stage, STS decreased macrophage foaming, lipid accumulation, and ROS levels. In advanced stages, IF reduces myocardial infarction and post-infarction cardiomyocyte hypertrophy, thereby promoting cardiac remodeling and survival^[49]^.

In conclusion, the current study demonstrated that IF inhibited acrolein-induced ROS-MAPK while activating AMPK and circadian clock gene expression, thereby ameliorating atherogenesis. Hence, IF represents a new therapeutic approach against environmental pollution-induced atherosclerosis.

However, there are still limitations in the current study. In the animal experiments, because of aggressive behaviors, one mouse died during the study; therefore, five mice per group were uniformly selected. Regarding experimental indicators, we focused on the development of early atherosclerosis in mice, such as the area of aortic plaques and levels of inflammation, and did not perform measurements of late plaque stability or assess the ratio of fibroblasts to collagen content. Additionally, due to technical issues, we did not extract mouse aortic progenitor cells and failed to fully characterize the cellular biological properties of atherosclerotic mice. Taken together, the AMPK-SIRT1/ROS-MAPK/CLOCK-BMAL1 regulatory axis may become a new target for the prevention and treatment of atherosclerosis in the future, pending validation by other investigators.

## SUPPLEMENTARY DATA

Supplementary data to this article can be found online.
