# Supplementary material for: Acrolein-induced atherosclerosis via AMPK/SIRT1-CLOCK/BMAL1 pathway and the protection from intermittent fasting
Source: J Biomed Res. 2024 May 29;39(6):549–63. doi: 10.7555/JBR.38.20240025 (PMC12683512; doi:10.7555/JBR.38.20240025)
Supplement: Supplementary file 1 — Supplementary data to this article can be found online. [file jbr-39-6-549-Supplementary.pdf]

Available online at www.jbr-pub.org.cn

Open Access at PubMed Central

JBR

Journal of Biomedical Research, 2025 39(6): 1–2

Supplementary Data

Acrolein-triggered atherosclerosis *via* AMPK/SIRT1-CLOCK/BMAL1 pathway and the protection from intermittent fasting

Qianfeng Chen[1,2,△], Yuxia Zhong[1,△], Bohan Li[1,△], Yucong Feng[1], Yuandie Zhang[1], Tao Wei[1], Margaret Zaitoun[1], Shuang Rong[3], Hua Wan[4,✉], Qing Feng[1,✉]

[1]*Department of Nutrition and Food Hygiene, Key Laboratory of Toxicology, School of Public Health, Nanjing Medical University, Nanjing, Jiangsu 211166, China;*
[2]*Department of Nosocomial Infection Management, Suzhou Wujiang District Children's Hospital, Suzhou, Jiangsu 215299, China;*
[3]*Department of Preventive Medicine, Department of Nutritional Health and Toxicology, School of Public Health, Wuhan University of Science and Technology, Wuhan, Hubei 430065, China;*
[4]*Healthcare Center, Sir Run Run Hospital, Nanjing Medical University, Nanjing, Jiangsu 211112, China.*

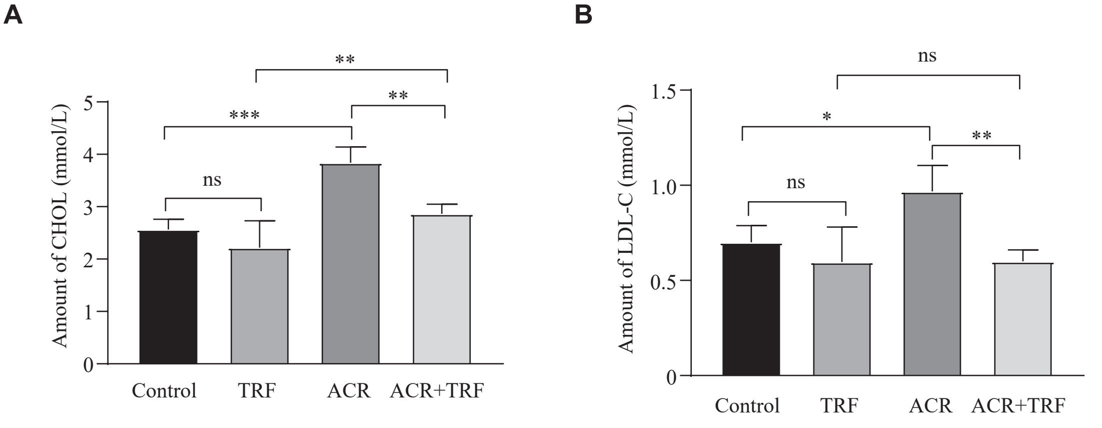

(Continued)

△These authors contributed equally to this work.

✉Corresponding authors: Hua Wan, Healthcare Center, Sir Run Run Hospital, Nanjing Medical University, 109 Longmian Avenue, Nanjing, Jiangsu 211112, China. E-mail: wanhua2006@njmu.edu.cn; Qing Feng, Department of Nutrition and Food Hygiene, Key Laboratory of Toxicology, School of Public Health, Nanjing Medical University, 101 Longmian Avenue, Nanjing, Jiangsu 211166, China. E-mail: qingfeng@njmu.edu.cn.

Received: 30 January 2024; Revised: 29 March 2024; Accepted: 30 April 2024; Published online: 29 May 2024

CLC number: R543, Document code: A

The authors reported no conflict of interests.

This is an open access article under the Creative Commons Attribution (CC BY 4.0) license, which permits others to distribute, remix, adapt and build upon this work, for commercial use, provided the original work is properly cited.

© 2025 by Journal of Biomedical Research.

https://doi.org/10.7555/JBR.38.20240025

(Continued)

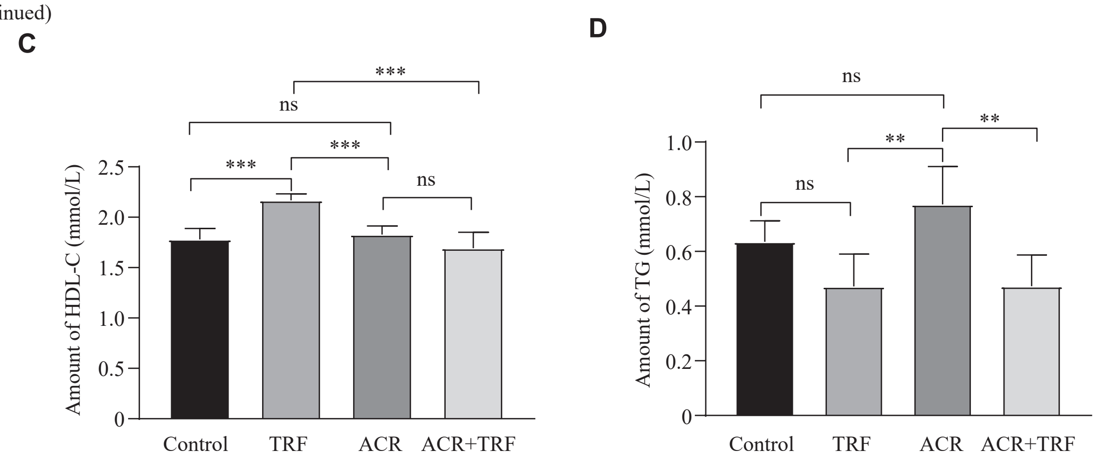

Supplementary Fig. 1 **Intervention effect of ACR and/or TRF on mice serum lipids by high-fat diet.** The C57BL/6J mice were fed a high-fat diet for eight weeks and then divided into four groups, including the Control, ACR, TRF, and ACR+TRF groups. These groups were treated with a high-fat diet, ACR plus high-fat diet, TRF (only eating a high-fat diet between 18:00 and 24:00), and ACR plus TRF for 12 weeks, respectively. The plasma was extracted from the eyeballs of mice, and the lipid levels were detected using a kit (n = 3). A: Amount of CHOL. B: Amount of LDL-C. C: Amount of HDL-C. D: Amount of TG. Data were expressed as mean ± standard deviation of at least three experiments and analyzed by one-way ANOVA, followed by the Bonferroni multiple comparison test. $^{*}P < 0.05$, $^{**}P < 0.01$, and $^{***}P < 0.001$. Abbreviations: CHOL, cholesterol; LDL-C, low-density lipoprotein cholesterol; HDL-C, high-density lipoprotein cholesterol; TG, triglyceride; ACR, acrolein; TRF, time-restricted fasting; ns, not significant.
